# Supplementary material for: Phase Angle Is Lower in Older Adults Living with HIV Compared to Geriatric Outpatients: A Case–Control Study
Source: J Clin Med. 2025 Aug 22;14(17):5941. doi: 10.3390/jcm14175941 (PMC12428825; doi:10.3390/jcm14175941)
Supplement: Supplementary file 1 [file jcm-14-05941-s001.zip › jcm-3815416-supplementary.pdf]

Supplement Table S1. Spearman correlation between phase angle and clinical parameters

	**Spearman r**	**p-value**
**Skeletal Muscle Index (SMI)**	0.42	< 0.001
**Handgrip Strength**	0.38	< 0.001
**Age**	−0.30	0.01
**Clinical Frailty Scale (CFS)**	−0.21	0.04
**Number of Medications**	−0.25	0.02
**Mini Nutritional Assessment–Short Form (MNA-SF)**	0.19	0.048
**Quick Mild Cognitive Impairment (Qmci)**	0.17	0.052

Spearman correlation analysis revealed that phase angle was positively correlated with skeletal muscle index (SMI) (r = 0.42, $p < 0.001$) and handgrip strength (r = 0.38, $p < 0.001$). A negative correlation was observed between phase angle and age (r = −0.30, $p = 0.01$), Clinical Frailty Scale (CFS) score (r = −0.21, $p = 0.04$), and number of medications (r = −0.25, $p = 0.02$). Weak but statistically significant positive correlations were also detected with Mini Nutritional Assessment Short Form (MNA-SF) score (r = 0.19, $p = 0.048$) and Quick Mild Cognitive Impairment (Qmci) score (r = 0.17, $p = 0.052$)(S1) .

Supplement Table S2. Diagnostic performance of phase angle in predicting HIV positivity (cut-off = 5.0)

Parameter	Value	95% Confidence Interval	p
**AUC**	0.69	0.58–0.80	0.004
**Cut-off value**	5.0	—	
**Sensitivity (%)**	15.0	—	
**Specificity (%)**	73.3	—	

*ROC analysis using phase angle to predict HIV status. At a cut-off of 5.0, specificity is relatively high while sensitivity is limited. AUC reflects moderate discriminative ability. This cut-off may be useful for confirmatory settings rather than screening.

Supplement 2 shows receiver operating characteristic (ROC) analysis was conducted to evaluate the discriminatory capacity of phase angle in identifying HIV-positive individuals. At a cut-off value of 5.0 degrees, the sensitivity and specificity were 15.1% and 73.3%, respectively, with an area under the curve (AUC) of 0.69 (95% CI: 0.58–0.80) and a statistically significant p-value (p = 0.004). Although the discriminatory power was moderate, the relatively low sensitivity at this cut-off suggests limited utility of phase angle as a standalone diagnostic marker. However, its moderate specificity may support its use as part of a broader clinical assessment, particularly in identifying individuals at higher risk of cellular or functional decline.

Supplement Table S3. Characteristics of people living with HIV

	n=50

**Duration of Infection, years,** median (IQR)	7.5 [7.5]
**CD4 levels((hücre/mm3),** median (IQR)	681.5 [448.5]
**CD8 levels (hücre/mm3),** median (IQR)	679.5 [644.75]
**CD4/CD8,** median (IQR)	0.79 [0.67]
**Medications**	
**ART drug number**, median (IQR)	3.0 [2.0]
**Non-HAART Users**, n (%)	-
**HAART Users,** n (%)	50 (100.0)
**Integraz Inhibitors,** n (%)	50 (100.0)
**NRTI,** n (%)	49 (98.0)
**NNRTI,** n (%)	1 (2.0)
**Boosters,** n (%)	2 (4.0)

ART, antiretroviral therapy; CD4,8, cluster of differentiation 4,8; HAART, highly active antiretroviral therapy; NNRTI, nonnükleozit reverse transcriptase inhibitor; NRTI: reverse transcriptase inhibitor

The baseline characteristics of the PLWH. The duration of infection was 7.5 years, and the CD4/CD8 ratio was 0.79 [0.67]. The median number of antiretroviral medications was 3.0 [2.0]; furthermore, most of the PLWH were on INSTI-based regimens, whereas one and two PLWH were on NNRTI- and bPI (boosted protease inhibitor)-containing regimens, respectively(S3).
